# Race, ethnicity, and language are underreported in hip fracture CPGs

**DOI:** 10.1007/s00402-026-06496-w

**Published:** 2026-09-18

**Authors:** Ashley Mulakaluri, Eli M. Snyder, Bill Young, Robin N. Kamal, Lauren M. Shapiro

**Affiliations:** 1 Keck School of Medicine, University of Southern California, Los Angeles, USA; 2 John A. Burns School of Medicine, University of Hawaii, Honolulu, USA; 3 VOICES Health Policy Research Center, Department of Orthopaedic Surgery, Stanford University, Stanford, USA; 4 Department of Orthopaedic Surgery, University of California, San Francisco, USA

**Keywords:** Hip fractures, Clinical practice guidelines, Patient-reported outcome measures, Sociodemographics

## Abstract

**Introduction:**

Clinical Practice Guidelines (CPGs) are systematically developed recommendations based on peer-reviewed literature that guide patient care. Considering the impact of sociodemographic factors on patient outcomes, this study aims (1) to review the racial, ethnic, and linguistic representation of patients in literature utilized to inform the CPGs for hip fractures in older adults and (2) to assess the use of linguistically- and culturally-adapted Patient Reported Outcome Measures (PROM) in these studies.

**Materials and methods:**

Publications listed under the American Academy of Orthopaedic Surgeons (AAOS) CPGs for the Management of Hip Fractures in Older Adults were identified and extracted. Variables extracted included: title, author, publication year, inclusion/exclusion criteria, country/ies of study, language of study, patient language, race and ethnicity, patient language proficiency, outcome measures utilized, translation or cultural-adaptation of outcome measures, and clinical trial registration page. The extracted variables were reported descriptively.

**Results:**

Of the 196 unique articles included, 12/196 (6.12%) reported race/ethnicity data and 9/196 (4.59%) reported patient language, of which 6/9 (66.7%) reported patient language proficiency. Of the studies that reported patient language or language proficiency, 8/9 (88.9%) specified this data in their inclusion or exclusion criteria. Of the 196 articles, 134/196 (67.4%) used one or more PROMs, and of these 7/134 studies (5.2%) reported the use of one or more translated PROMs. Of these, 4/7 studies (57.1%) reported the use of one or more culturally-adapted PROMs.

**Conclusions:**

This study found a paucity of reporting of patient language, race, and ethnicity in the studies utilized to inform the AAOS CPGs for hip fractures in elderly adults. Additionally, there is minimal usage of translated and/or culturally-adapted PROMs in these studies. As care outcomes are associated with sociodemographic factors, improved reporting of these factors in guideline-informing literature may enhance guideline applicability and health equity.

**Level of Evidence:**

V.

## Introduction

Hip fractures, particularly those in the elderly, are a prevalent public health issue globally. Hip fractures have increasingly been shown to be a major cause of premature mortality and morbidity in the elderly, especially with the rising life expectancy [1–3]. As most patients are admitted to the hospital for the surgical treatment of hip fractures, there are many decisions that are made across the care process (pre-operatively, peri-operatively, post-operatively). These include decisions such as surgical approach, anesthetic type, and utilization of thromboembolic prophylaxis [3]. The specifics of each decision are dependent on patient characteristics and should be guided by the best available evidence with the goal of ensuring high quality care.

Clinical Practice Guidelines (CPGs) are systematically developed recommendations based on best-available, peer-reviewed studies. CPGs help practitioners and patients better understand the associated condition and make informed decisions according to patient- and injury-specific characteristics. CPGs serve the role of facilitating physician-patient dialogue by presenting evidence-based recommendations on management of care [4]. The American Academy of Orthopaedic Surgeons (AAOS), an organization whose goal is to advocate for and provide high-quality musculoskeletal care and education, develops CPGs in a standardized method to help promote the delivery of high-quality care. Patient Reported Outcome Measures (PROM), questionnaires aimed at understanding patient symptoms, functionality, and quality of life, are often utilized as endpoints/outcome measures for clinical outcome studies, especially in orthopaedics [5, 6, 7]. PROM use is recommended to better understand patient priorities and outcomes and helps facilitate physician-patient dialogue to promote patient-centered care [5].

Many patient-specific and sociodemographic factors have known associations with outcomes, including PROM scores. Sociodemographic factors, such as language, race, and ethnicity have been associated with differing outcomes in patients with hip fractures [8–12]. For example, Penrod et al. found higher survival (odds ratio of 1.74) and improved mobility (odds ratio of 1.54) in white patients compared to non-white patients after a hip fracture [13]. Similarly, Nayar et al. found differences in time to surgery and perioperative complications—such as reintubation and pulmonary embolism—among patients of different races with hip fractures, with African Americans having the worst outcomes compared to white patients and Asian patients except for mortality [14]. When considering PROMs, Cohen-Levy et al. found non-white race/ethnicity was associated with lower Patient Reported Outcomes Measurement Information System (PROMIS) - Mental Health scores and Knee Injury and Osteoarthritis Outcome Score (KOOS) scores, when undergoing total hip arthroplasty [15]. With regard to language, in a total joint arthroplasty cohort, Manuel et al. demonstrated that limited English proficiency was independently associated with longer length of stay and discharge to skilled care [16]. As patients with differing sociodemographic factors may have differing outcomes, the reporting of such factors in studies may help contextualize outcomes in a patient-centered manner. Additionally, doing so may enhance the applicability of the CPGs informed by such literature, particularly as the United States population becomes more diverse [17, 18]. As such, this study aims (1) to review the racial, ethnic, and linguistic representation of patients in the literature cited by the CPGs for hip fractures in older adults and (2) to assess the use of linguistically- and culturally-adapted PROMs in these studies.

## Methods

### Article identification and inclusion

Two reviewers (AM, ES) independently identified publications listed under the Clinical Practice Guidelines for the Management of Hip Fractures in Older Adults for inclusion based on their citation by the AAOS CPGs website [19]. If the clinical trial registration page was available for a study, the page was evaluated as well.

The following variables were collected by the authors (AM, ES, BY) in a standardized excel form (Microsoft Inc, Seattle, USA): title, author, publication year, journal, type of study, country/ies of study, inclusion and exclusion criteria, diagnosis, patient race and ethnicity (if reported), language of study, patient language or proficiency (if reported), PROMs utilized (if reported), and report of translation or cultural-adaptation of outcome measures. The extracted variables were reported descriptively. Discrepancies were resolved by consensus between reviewers. PROM use was determined by if the study had explicitly reported a specific PROM instrument or if listed under the corresponding clinical trial registration page. Translated PROMs were defined as those adapted from an original language to a target language and validated to have the same meaning [20, 21]. Culturally-adapted PROMs are those adjusted to account for cultural differences (not just linguistic or translation differences) in interpretation of the PROM content while maintaining content validity [20, 21].

## Results

The AAOS CPGs for hip fractures in older adults has 213 citations of which 17 duplicates were removed. One hundred and ninety-six studies were extracted and data was collected in a standardized data collection form by three authors (AM, ES, BY).

### Patient race and ethnicity data

Of the 196 articles included, 12/196 (6.12%) reported race/ethnicity data. Of the studies that reported participant race/ethnicity data, 9/12 (75.0%) had a majority White/Caucasian participant population, with 6/9 (66.7%) reporting above 90% White/Caucasian participants of the total participant pool (Table 1). The other three studies reported a participant population of “100% Taiwanese” and were conducted in Taiwan.

**Table 1:** Studies reporting race/ethnicity, language, and/or use of translated patient reported outcome measures (PROMs) – as reported

First author	Year	Journal	Country of study	Race/Ethnicity reported	Reported language of participants	PROMs collected	Translated PROM use and language
Berggren [61]	2008	Osteoporosis International	Sweden	NR	NR	GDS-15, ADL, S-COVS	Swedish COVS
Bhandari [62]	2019	New England Journal of Medicine	USA, Canada, Spain, UK, Netherlands, Norway, Finland, Australia, New Zealand, South Africa	Native or aboriginal (0.21%), South Asian (0.63%), East Asian (0.97%), Hispanic or Latino (0.9%), White (95.13%), Black (1.88%), Middle Eastern (0.28%)	NR	NR	NR
Carson [63]	2015	Lancet	USA, Canada	White (93.8%)	NR	NR	NR
Carson [64]	2011	New England Journal of Medicine	USA, Canada	White (93.8%), Black (4.07%), Asian (1.34%), Other (0.79%)	NR	NR	NR
Clemmesen [65]	2018	Anaesthesia	Denmark	NR	Danish	NR	NR
Deangelis [66]	2012	Journal of Orthopaedic Trauma	USA	White (93.85%)	NR	NR	NR
Gregersen [67]	2015	Journal of American Medical Directors Association	Denmark	NR	Danish	NR	NR
Gruber-Baldini [68]	2013	Journal of the American Geriatrics Society	USA, Canada	White (73.2%), Black (8.7%), Unspecified (0.72%)	NR	NR	NR
Keating [69]	2005	Health Technology Assessment	UK	NR	English	NR	NR
Kenzora [70]	1998	Clinical Orthopaedics and Related Research	USA	White (89.6%), Other (10.4%)	NR	NR	NR
Marcantonio [71]	2001	Journal of the American Geriatrics Society	USA	Caucasian (90.48%)	NR	NR	NR
Morrison [72]	2016	Journal of the American Geriatrics Society	USA	White (90.2%), Black (5.2%), Other (4.58%), Non-Hispanic (94.12%)	English, Spanish, or Russian	NR	NR
Radcliff [73]	2008	Journal of Bone & Joint Surgery	USA	White (76%), Black (12%), Other (13%)	NR	NR	NR
Shyu [74]	2010	Journal of American Geriatrics Society	China	100% Taiwanese	NR	BI	Chinese BI
Shyu [75]	2013	The Journal of Gerontology: Series A	China	100% Taiwanese	NR	BI, GDS	Chinese BI, Chinese GDS
Shyu [76]	2013	International Journal of Nursing Studies	China	NR	NR	BI score, SF-36, GDS	Chinese BI, Chinese SF-36, Chinese GDS
Shyu [77]	2016	International Journal of Nursing Studies	China	100% Taiwanese	NR	Chinese BI, IADL performance	Chinese BI, Chinese IADL performance
Tosun [78]	2018	International Journal of Orthopaedic and Trauma Nursing	Turkey	NR	Turkish	VAS pain, VAS satisfaction, ICQ	Turkish ICQ
Tzimas [79]	2018	Injury	Greece	NR	Greek	NR	NR
Van de Bekerom [80]	2010	Journal of Bone & Joint Surgery	Netherlands	NR	Dutch	NR	NR
Wei [81]	2020	Journal of the American Geriatrics Society	China	NR	NR	HHS, EQ-5D, VAS pain	Chinese EQ-5D
Yip [82]	2002	International Orthopaedics	China	NR	“Chinese language” as stated in text.	NR	NR

Abbreviation *ADL* Activities of Daily Living, *BI* Barthel Index, *EQ-5D* EuroQol-5 Dimension, *GDS* Geriatric Depression Scale, *HHS* Harris Hip Score, *IADL* Instrumental Activities of Daily Living, *ICQ* Immobilization Comfort Questionnaire, *NR* Not reported, *S-COVS* Swedish-Clinical Outcome Variables Scale, *SF-36* Short Form-36, *VAS* Visual Analog Scale

### Patient language data

Of the articles analyzed, 9/196 (4.59%) reported patient language and of these, 6/9 (66.7%) reported patient language proficiency. Of note, one study excluded patients with “language problems”, however, did not specify patient language, what they defined as language proficiency or what a “language problem” may be, and hence was excluded from the total [22]. The studies reporting patient language or language proficiency showed 8/9 (88.9%) specified this data in their inclusion or exclusion criteria. Among the studies that reported patient language data, English was the most frequently reported language in 3/9 (33.3%) of studies. The other languages reported were Danish (2/9, 22.2%), Spanish (1/9, 11.1%), Russian (1/9, 11.1%), Turkish (1/9, 11.1%), Greek (1/9, 11.1%), Dutch (1/9, 11.1%), and Chinese (1/9, 11.1%). Morrison et al. was the only study with multiple reported patient languages (English, Spanish, and Russian).

Across the 196 studies, 37 different countries of investigation were reported (note that the UK was reported separately from England, Scotland, and Ireland). There were 5 multinational studies, only one of which—with study locations in the United States and Canada—reported patient language.

### The use of translated or culturally-adapted PROMs

Of the total studies, 134/196 (67.4%) used one or more PROMs as a study end point. Of the 134, 7/134 studies (5.2%) reported the use of one or more translated PROMs (Table I). Of these, 4/7 studies (57.1%) reported the use of one or more culturally-adapted PROMs. Across the multinational studies and/or those that reported the inclusion of patients with non-English languages, 1/12 (8.3%) utilized translated PROMs.

### Clinical trial registration page findings

Clinical Trial Registration numbers were identified in 38 studies. Examples of reported registries included ClinicalTrials.gov, International Standard Randomized Controlled Trial Number (ISRCTN) Registry, EU Clinical Trials Register, Australian New Zealand Clinical Trials Registry (ANZCTR), and Chinese Clinical Trials Register (ChiCTR). Of the clinical trial registration numbers available, three pages (7.9%) were not accessible.

In summary (of the data extracted from the literature and the clinical trial registration pages), most studies (95%) did not report language data including patient language nor proficiency, nor did most (92%) report race/ethnicity data. Finally, most studies that used one or more PROMs (95%) did not report the use translated or culturally-adapted PROMs.

## Discussion

This review illustrates a paucity of reporting of patient race, ethnicity, and language data in the literature cited by the AAOS CPGs for hip fractures in the elderly. As such sociodemographic factors have been linked to differing outcomes in clinical settings, the lack of reporting of such factors in the studies informing CPGs may potentially limit CPGs generalizability and application [23]. Additionally, as CPGs help facilitate understanding of a particular condition and associated treatment among physicians and patients, their limited applicability may weaken patient-physician dialogue and shared decision-making. Of note, this study was descriptive and cannot make conclusions regarding the causal relationship between sociodemographic factors and clinical outcomes. However, without the reporting of such sociodemographic factors and broad inclusion of diverse patients, guideline-informing literature may inadequately encompass diversity in orthopaedics, particularly in hip fracture care, which may contribute to healthcare inequities.

Similar to our findings, previous studies have highlighted the underrepresentation of traditionally minoritized groups and limited reporting of sociodemographic data in clinical trials [24–28]. However, when considering sex/gender in particular, there has not only been an increase in standardization of reporting of sex/gender but also stratifying data analysis according to these variables for optimal patient care [29–33]. Concordantly, Alegria et al. found participant sex/gender data was increasingly reported when comparing randomized clinical trials from 2019 to those published in 2015 [24]. Increased reporting of sex/gender may have helped identify gaps in the research on underrepresented participant pools, helping drive broader recruitment and inclusion for future studies. Of note, in this same study, Alegria et al. found race data was still lacking [24]. Patient sociodemographic factors including race and ethnicity data have been linked to differences in patient outcomes. For example, Jarman et al. found non-Hispanic Black hip fracture patients had not only an increased risk of post-operative complications (e.g. urinary tract infections, deep vein thrombosis, ulcers, and embolisms) and but also longer length of stay [34]. Likewise, Oduguwa et al. found Black patients had increased costs of hip fracture treatment, and increased rates of complications compared to White patients [35]. Such differences have been reported in a variety of clinical studies on patients with hip fractures when sociodemographic variables, like race, are included [13, 36–38]. Clinical results and guidelines are most applicable to those in whom they were investigated and developed. Therefore, the inclusion of diverse populations is warranted for the results and guidelines to be applicable in those populations. As such, it is important to not only report such data in the literature, but to promote inclusion and diversity in investigation. Our study found a similar paucity in the reporting of race, ethnicity, and language information (e.g., rate) as well as diversity of the participant pool reported in the studies informing the CPGs. For example, of the 9 studies included that were conducted in the United States that reported race and ethnicity data, the average percent of white patients was 88.45%, greatly exceeding the national average (61.6%) according to the 2020 US Census Bureau [18]. These findings are similar to a study by Gilliam et al. investigating pediatric CPGs; of the CPGs reviewed, they found 70% did not mention race [39]. By expanding and standardizing patient demographic reporting to include race, ethnicity, and language data, researchers can be more cognizant of which demographics are underrepresented. By doing so, future studies may focus on conducting research with a more diverse patient cohort that facilitates the broad applicability of findings to underrepresented patient populations. Notably, some journals and research institutions have recommended guidelines for the inclusion and reporting of patients’ demographic information to improve transparency and understanding of differences based upon such variables [30, 40–45]. The collection and reporting of patient race and ethnicity data could be more widely adopted by research investigations to promote equity and improved understanding of outcome differences based upon such variables.

It is important to acknowledge that race and ethnicity should not be considered in isolation, but rather in the context of broader sociodemographic factors and social determinants of health [44]. Furthermore, limited diversity with respect to participants in clinical trials could be due to unwillingness of traditionally minoritized communities to participate in health research. This may be due to cultural and linguistic differences, time and financial constraints, or fear of exploitation due to past experiences [46, 47]. To better understand race, ethnicity, and language differences among patients and the impact of these factors on their health outcomes, it is important for researchers to acknowledge the underrepresentation of traditionally minoritized communities in studies. Furthermore, researchers may then identify the barriers to access to care and recruitment in these groups and generate strategies to overcome these barriers. Standardizing collection of race, ethnicity, and language data may help with interpreting data applicability to specific patient cohorts, and allow researchers to identify which groups are being underrepresented in their investigation. Additionally, use of appropriate research tools catered towards a more diverse participant cohort, such as translated and culturally-adapted PROMs, may facilitate diverse participant recruitment and retention within studies. Such strategies help minimize bias in research, facilitate diversity in study participants, and thereby improve the applicability of study results to the general population.

Patient language and language proficiency have been linked to differing outcomes in pre-, peri-, and post-operative care, hence we sought to understand the extent to which it is reported and considered in the CPG-informing literature. Patient language has been demonstrated to be associated with complications and outcomes [48, 49]. Therefore, reporting patient language as a variable in guideline informing literature, as well as including patients with diverse linguistic preferences with appropriate accommodations (e.g., interpreter, translated materials) in the study is important [48, 49]. For example, Reppas-Rindlisbacher et al. demonstrated that patients with a hip fracture with a preference for a non-English language had a greater rate of complications (e.g. myocardial infarction, delirium) and longer hospital stays [50]. Konopka et al. demonstrated non-English-speaking patients had significantly lower PROM scores preoperatively and postoperatively for those undergoing total joint arthroplasty [51]. Similarly, Paz et al. found that patients that spoke different languages responded differently on PROMs, despite having similar underlying physical function [8]. Our study found 95% of articles did not report any patient language or proficiency data. As studies have shown differences in outcomes between patients who speak different languages, it is important to consider such sociodemographic factors in study interpretation, and as an extension CPGs [8, 50, 51]. Sociodemographic information, including language, should be recorded during participant intake for studies, such as through an intake survey. Additionally, validated measures of language proficiency may provide more detailed language data while also helping to facilitate patient-centered care. At the level of the CPGs development process, articles of all languages should be considered, as the current process only includes articles written in English [52]. CPGs may benefit from transparent reporting of potential limitations in their article selection process. Additionally, CPGs currently preface each recommendation with a brief summary with level of evidence; including a patient-targeted summary explaining the applicability of their recommendation to specific demographics can help inform patients’ decision-making.

PROMs are an increasingly common method by which to facilitate patient-centered care that can identify individual patient priorities and facilitate shared decision-making [5, 53, 54]. As supported by Konopka et al. and Paz et al., failure to utilize and report appropriately translated or culturally-adapted PROMs may limit interpretation of patient outcomes [8, 51, 55, 56]. In this study, use of appropriately translated and culturally-adapted PROMs was only assessed among studies that reported collecting PROMs (134/196). Our study revealed, despite a majority of the studies utilizing PROMs, only 5% reported the use of translated or culturally-adapted PROMs. Of note, this does not imply that appropriately translated or culturally-adapted PROMs were not offered as this conclusion cannot be drawn. Translated and culturally-adapted PROMs have become increasingly available for use [8, 57, 58]. Therefore, the use of translated and culturally-adapted PROMs is an opportunity to increase the inclusion and diversity of clinical trials and can help minimize the barriers posed by language and comprehension differences [59]. Konopka et al. found that non-English-speaking, ethnic, and socioeconomic minorities were less likely to fill out PROMs [51]. This is especially important to consider in the US, where 22.5% of individuals reported speaking a language other than English, of whom 38.8% reported speaking English “less than very well” [60]. Clinically, appropriate use of translated and culturally-adapted PROMs allows for accurate capture of patient outcomes which may improve patient-centered clinical decision-making and assessment of treatment effectiveness. Academically, appropriate use of PROMs may facilitate diversity and inclusion in research as well as increase patient retention for studies.

This study has limitations. The screening methodology of this study poses a risk of heterogeneity in data collection among multiple reviewers. We minimized this risk through strict inclusion criteria and cross-checking of data collection between reviewers to ensure consistency. Additionally, the investigators of this study did not confirm with nor contact any of the authors of the studies to evaluate the collection or reporting of sociodemographic information. It is possible that the studies did collect data on patient language, race, or ethnicity and did not report this information in the manuscript or trial registration page. For example, it is possible that the PROM utilized by the study was language concordant with the study population. However, as most studies did not report patient language nor PROM language, no conclusions can be drawn in this regard. Furthermore, the study investigators did not verify whether the appropriately translated or culturally-adapted PROMs were available to the individual studies but not reported, which may not accurately represent the availability of appropriately adapted instruments. These findings solely aim to comment on the reporting practices of guideline-informing literature. Regarding the inclusion criteria for the study, the review solely focused on articles cited by the AAOS CPGs for hip fractures in older adults—the findings may not be applicable to broader hip fracture literature. Finally, we acknowledge that race, ethnicity, and language are factors that coexist in a larger context of sociodemographic variables which deserve investigation as well. For example, the consent refusal rate for study participation was not investigated which may introduce selection bias if specific demographic groups were more likely to refuse.

## Conclusions

Overall, there is a paucity in reporting of patient race, ethnicity, and language in the studies informing the AAOS CPGs for hip fractures in elderly adults. Additionally, there is minimal reporting of the use of translated or culturally-adapted PROMs in these studies. The diversity and inclusion of orthopaedic research should parallel the vast and increasing diversity of the orthopaedic patient population. Hence, when utilizing such guidelines to plan patient care, clinicians should interpret current recommendations with the awareness that their conclusions may not be applicable to diverse patient populations as current guideline-supporting literature has been shown to underreport sociodemographic variables. Improved reporting of such variables may help enhance external validity of study findings and therefore guidelines themselves. Given the association between care outcomes and sociodemographic factors, consistent transparent reporting of these factors may increase guideline generalizability and applicability while helping to facilitate improved healthcare quality and equity.

## Data Availability

No datasets were generated or analysed during the current study.
